# 3-(1-Ethyl-1*H*-pyrrole-2-carboxamido)propionic acid monohydrate

**DOI:** 10.1107/S1600536809026749

**Published:** 2009-07-15

**Authors:** Dong Dong Li, Gui Hong Tang, Xiang Chao Zeng, Gang Huang, Xing Yan Xu

**Affiliations:** a Department of Chemistry, Jinan University, Guangzhou, Guangdong, 510632, People’s Republic of China

## Abstract

The title compound, C_10_H_14_N_2_O_3_·H_2_O, was synthesized by alkyl­ation of methyl 3-(1*H*-pyrrole-2-carboxamido)­propion­ate with ethyl bromide, followed by saponification and acidification. In the crystal structure, inter­molecular O—H⋯O and N—H⋯O hydrogen bonds link the mol­ecules, forming layers parallel to the *ac* plane.

## Related literature

For pyrroles sourced from marine organisms, see: Liu *et al.* (2005 ▶). For the bioactivity of pyrrole derivatives, see: Banwell *et al.* (2006 ▶); Sosa *et al.* (2002 ▶). For related structures, see: Zeng *et al.* (2005 ▶); Liu *et al.* (2006 ▶); Tang *et al.* (2008 ▶).
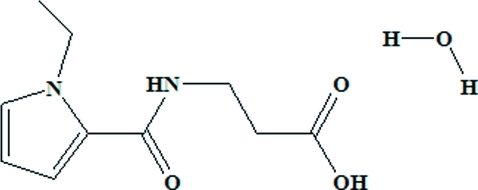


## Experimental

### 

#### Crystal data

C_10_H_14_N_2_O_3_·H_2_O
*M* *_r_* = 228.25
Monoclinic, 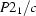

*a* = 5.2814 (12) Å
*b* = 31.795 (7) Å
*c* = 7.0226 (16) Å
β = 106.392 (4)°
*V* = 1131.3 (4) Å^3^
*Z* = 4
Mo *K*α radiation
μ = 0.10 mm^−1^
*T* = 173 K
0.47 × 0.44 × 0.15 mm

#### Data collection

Bruker SMART 1K CCD area-detector diffractometer
Absorption correction: multi-scan (*SADABS*; Sheldrick, 1996 ▶) *T*_min_ = 0.953, *T*_max_ = 0.985
5260 measured reflections
2215 independent reflections
1772 reflections with *I* > 2σ(*I*)
*R*_int_ = 0.028

#### Refinement

*R*[*F* ^2^ > 2σ(*F* ^2^)] = 0.052
*wR*(*F* ^2^) = 0.163
*S* = 1.14
2215 reflections
155 parameters
H atoms treated by a mixture of independent and constrained refinement
Δρ_max_ = 0.30 e Å^−3^
Δρ_min_ = −0.26 e Å^−3^

### 

Data collection: *SMART* (Bruker,1999 ▶); cell refinement: *SAINT-Plus* (Bruker, 1999 ▶); data reduction: *SAINT-Plus*; program(s) used to solve structure: *SHELXS97* (Sheldrick, 2008 ▶); program(s) used to refine structure: *SHELXL97* (Sheldrick, 2008 ▶); molecular graphics: *SHELXTL* (Sheldrick, 2008 ▶); software used to prepare material for publication: *SHELXTL*.

## Supplementary Material

Crystal structure: contains datablocks I, global. DOI: 10.1107/S1600536809026749/rz2348sup1.cif

Structure factors: contains datablocks I. DOI: 10.1107/S1600536809026749/rz2348Isup2.hkl

Additional supplementary materials:  crystallographic information; 3D view; checkCIF report

## Figures and Tables

**Table 1:** Hydrogen-bond geometry (Å, °)

*D*—H⋯*A*	*D*—H	H⋯*A*	*D*⋯*A*	*D*—H⋯*A*
O3—H3⋯O4^i^	0.84	1.83	2.669 (3)	173
N2—H2⋯O4^ii^	0.88	2.28	3.091 (3)	154
O4—H4*A*⋯O1^iii^	0.96 (3)	1.79 (3)	2.737 (2)	170 (3)
O4—H4*B*⋯O2^iv^	0.81 (3)	2.08 (4)	2.863 (3)	164 (3)

Symmetry codes: (i) 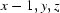; (ii) 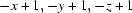; (iii) 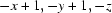; (iv) 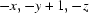.

**Fig. 1.**
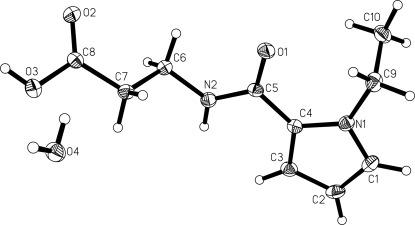
The molecular structure of the title compound, with the atom-numbering scheme. Displacement ellipsoids are drawn at the 30% probability level.

**Fig. 2.**
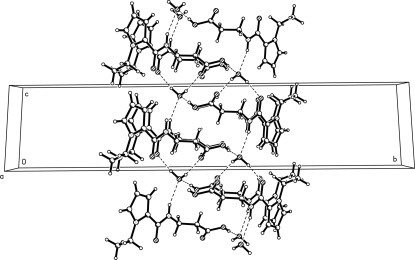
Crystal packing of the title compound viewed along the a axis. Dashed lines indicate hydrogen bonds.

**Fig. 3.**
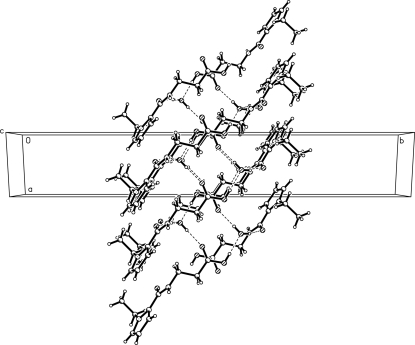
Crystal packing of the title compound viewed along the c axis. Dashed lines indicate hydrogen bonds.

## supplementary crystallographic information

### Comment

Pyrrole derivatives are well known constituents of many marine organisms (Liu *et al.*, 2005), and some of them show important bioactivities, such as antitumor (Banwell *et al.*, 2006) and protein kinase inhibiting (Sosa *et al.*, 2002) activities. As a continuation of our studies in this field, which have recently resulted in the communication of the crystal structure of 3-(4-bromo-1*H*-pyrrole-2-carboxamido)propanoic acid (Zeng *et al.*, 2005), 3-(1-methyl-1H-pyrrole-2-carboxamido)propanoic acid (Liu *et al.*, 2006) and methyl 2-(1*H*-pyrrole-2-carboxamido)acetate (Tang *et al.*, 2008), we report herein the synthesis and crystal structure of the title compound.

In the molecule of the title compound (Fig. 1), bond lengths and angles are unexceptional. In the crystal structure, molecules are linked by intermolecular O—H···O and N—H···O hydrogen bonds (Table 1) involving water molecules to form two-dimensional layers parallel to the *ac* plane (Fig. 2, Fig. 3)

### Experimental

A suspension of potassium carbonate (2.10 mg, 15.0 mmol), ethyl bromide (1.87 ml, 25.0 mmol) and methyl 3-(1*H*-pyrrole-2-carbonyl)aminopropionate (0.98 g, 5.0 mmol) in acetonitrile (12 ml) was refluxed for 40 h. After evaporation of the solvent, the residue was dissolved in ethyl acetate (15 ml) and washed twice with water. The organic layer was dried over sodium sulfate and evaporated *in vacuo*. Then the alkylated product was added to a solution of 10% aqueous sodium hydroxide (10 ml) and ethanol (2 ml), and the mixture was stirred at room temperature for 24 h. The hydrolyzed mixture was made acidic with 10% hydrochloric acid to pH 2–3. After filtration, the precipitate was collected as a yellow solid (m.p. 320 K, 92.3% yield). Pale yellow crystals suitable for X-ray analysis were obtained over a period of one week by slow evaporation at room temperature of an ethanol/water solution (3:2 v/v).

### Refinement

All non-H atoms were refined with anisotropic displacement parameters. The water H atoms were located in a difference Fourier map and refined freely. All other H atoms were positioned geometrically and refined using a riding model, with C—H = 0.95-0.99Å, N—H = 0.88 Å, O—H = 0.84 Å, and with *U*_iso_ = 1.2 *U*_eq_(C, N) or 1.5 *U*_eq_(C, O) for methyl and hydroxy H atoms.

### Crystal data

**Table:**

C_10_H_14_N_2_O_3_·H_2_O	*F*(000) = 488
*M**_r_* = 228.25	*D*_x_ = 1.340 Mg m^−^^3^
Monoclinic, *P*2_1_/*c*	Melting point: 320 K
Hall symbol: -P 2ybc	Mo *K*α radiation, λ = 0.71073 Å
*a* = 5.2814 (12) Å	Cell parameters from 2595 reflections
*b* = 31.795 (7) Å	θ = 2.6–28.0°
*c* = 7.0226 (16) Å	µ = 0.10 mm^−^^1^
β = 106.392 (4)°	*T* = 173 K
*V* = 1131.3 (4) Å^3^	Plate, pale yellow
*Z* = 4	0.47 × 0.44 × 0.15 mm

### Data collection

**Table:**

Bruker SMART 1K CCD area-detector diffractometer	2215 independent reflections
Radiation source: fine-focus sealed tube	1772 reflections with *I* > 2σ(*I*)
graphite	*R*_int_ = 0.028
φ and ω scans	θ_max_ = 26.0°, θ_min_ = 1.3°
Absorption correction: multi-scan (*SADABS*; Sheldrick, 1996)	*h* = −6→4
*T*_min_ = 0.953, *T*_max_ = 0.985	*k* = −39→33
5260 measured reflections	*l* = −8→8

### Refinement

**Table:**

Refinement on *F*^2^	Primary atom site location: structure-invariant direct methods
Least-squares matrix: full	Secondary atom site location: difference Fourier map
*R*[*F*^2^ > 2σ(*F*^2^)] = 0.052	Hydrogen site location: inferred from neighbouring sites
*wR*(*F*^2^) = 0.163	H atoms treated by a mixture of independent and constrained refinement
*S* = 1.14	*w* = 1/[σ^2^(*F*_o_^2^) + (0.087*P*)^2^ + 0.3989*P*] where *P* = (*F*_o_^2^ + 2*F*_c_^2^)/3
2215 reflections	(Δ/σ)_max_ = 0.001
155 parameters	Δρ_max_ = 0.30 e Å^−^^3^
0 restraints	Δρ_min_ = −0.26 e Å^−^^3^

### Special details

**Table:**

Geometry. All e.s.d.'s (except the e.s.d. in the dihedral angle between two l.s. planes) are estimated using the full covariance matrix. The cell e.s.d.'s are taken into account individually in the estimation of e.s.d.'s in distances, angles and torsion angles; correlations between e.s.d.'s in cell parameters are only used when they are defined by crystal symmetry. An approximate (isotropic) treatment of cell e.s.d.'s is used for estimating e.s.d.'s involving l.s. planes.
Refinement. Refinement of *F*^2^ against ALL reflections. The weighted *R*-factor *wR* and goodness of fit *S* are based on *F*^2^, conventional *R*-factors *R* are based on *F*, with *F* set to zero for negative *F*^2^. The threshold expression of *F*^2^ > σ(*F*^2^) is used only for calculating *R*-factors(gt) *etc*. and is not relevant to the choice of reflections for refinement. *R*-factors based on *F*^2^ are statistically about twice as large as those based on *F*, and *R*- factors based on ALL data will be even larger.

### Fractional atomic coordinates and isotropic or equivalent isotropic displacement parameters (Å^2^)

**Table:**

	*x*	*y*	*z*	*U*_iso_*/*U*_eq_	
O2	−0.2174 (3)	0.48016 (5)	0.1312 (3)	0.0377 (4)	
O1	0.4540 (3)	0.36651 (5)	0.1693 (2)	0.0350 (4)	
N1	0.9087 (4)	0.32234 (6)	0.4136 (3)	0.0281 (5)	
O3	0.0271 (4)	0.53444 (5)	0.2775 (3)	0.0370 (5)	
H3	−0.1105	0.5479	0.2213	0.055*	
N2	0.3901 (4)	0.39704 (6)	0.4423 (3)	0.0307 (5)	
H2	0.4458	0.3994	0.5723	0.037*	
C4	0.7462 (4)	0.34863 (6)	0.4836 (3)	0.0254 (5)	
C6	0.1614 (4)	0.42138 (7)	0.3356 (3)	0.0295 (5)	
H6A	0.0205	0.4183	0.4023	0.035*	
H6B	0.0936	0.4103	0.1990	0.035*	
C5	0.5200 (4)	0.37122 (6)	0.3528 (3)	0.0250 (5)	
C7	0.2283 (4)	0.46754 (7)	0.3265 (3)	0.0277 (5)	
H7A	0.3132	0.4778	0.4627	0.033*	
H7B	0.3567	0.4707	0.2485	0.033*	
C8	−0.0108 (5)	0.49402 (7)	0.2343 (3)	0.0267 (5)	
C3	0.8518 (5)	0.35132 (7)	0.6881 (3)	0.0319 (6)	
H3A	0.7824	0.3673	0.7761	0.038*	
C9	0.8797 (5)	0.30933 (8)	0.2082 (3)	0.0361 (6)	
H9A	1.0516	0.2987	0.1980	0.043*	
H9B	0.8319	0.3342	0.1206	0.043*	
C1	1.1074 (5)	0.30880 (8)	0.5705 (4)	0.0350 (6)	
H1	1.2446	0.2901	0.5629	0.042*	
C10	0.6725 (6)	0.27552 (8)	0.1358 (4)	0.0407 (6)	
H10A	0.7317	0.2493	0.2084	0.061*	
H10B	0.6457	0.2708	−0.0065	0.061*	
H10C	0.5060	0.2846	0.1585	0.061*	
C2	1.0782 (5)	0.32635 (8)	0.7411 (4)	0.0366 (6)	
H2A	1.1912	0.3223	0.8715	0.044*	
O4	0.5768 (4)	0.57638 (6)	0.1280 (3)	0.0343 (4)	
H4A	0.584 (6)	0.5980 (10)	0.034 (5)	0.057 (9)*	
H4B	0.465 (7)	0.5596 (10)	0.074 (5)	0.051 (9)*	

### Atomic displacement parameters (Å^2^)

**Table:**

	*U*^11^	*U*^22^	*U*^33^	*U*^12^	*U*^13^	*U*^23^
O2	0.0288 (9)	0.0372 (9)	0.0415 (10)	0.0021 (7)	0.0009 (8)	−0.0031 (7)
O1	0.0450 (10)	0.0355 (9)	0.0196 (8)	0.0068 (8)	0.0013 (7)	−0.0001 (6)
N1	0.0253 (10)	0.0322 (10)	0.0270 (10)	−0.0003 (8)	0.0077 (8)	0.0016 (7)
O3	0.0413 (11)	0.0273 (8)	0.0404 (10)	0.0022 (7)	0.0087 (8)	−0.0014 (7)
N2	0.0350 (11)	0.0324 (10)	0.0220 (9)	0.0048 (8)	0.0038 (8)	0.0004 (8)
C4	0.0285 (12)	0.0239 (10)	0.0227 (11)	−0.0027 (9)	0.0057 (9)	0.0011 (8)
C6	0.0255 (12)	0.0301 (12)	0.0309 (12)	0.0011 (9)	0.0048 (9)	−0.0013 (9)
C5	0.0290 (11)	0.0225 (10)	0.0222 (11)	−0.0043 (9)	0.0049 (9)	0.0010 (8)
C7	0.0255 (11)	0.0311 (12)	0.0265 (11)	−0.0032 (9)	0.0076 (9)	−0.0026 (9)
C8	0.0311 (12)	0.0292 (11)	0.0219 (10)	−0.0009 (9)	0.0110 (9)	−0.0003 (8)
C3	0.0376 (13)	0.0312 (12)	0.0235 (11)	−0.0002 (10)	0.0029 (10)	0.0005 (9)
C9	0.0368 (14)	0.0459 (14)	0.0306 (12)	0.0049 (11)	0.0179 (11)	0.0016 (10)
C1	0.0258 (12)	0.0371 (13)	0.0393 (13)	0.0020 (10)	0.0044 (10)	0.0052 (10)
C10	0.0506 (16)	0.0392 (14)	0.0304 (13)	0.0054 (12)	0.0082 (12)	−0.0061 (10)
C2	0.0341 (13)	0.0394 (14)	0.0282 (12)	−0.0038 (11)	−0.0044 (10)	0.0039 (10)
O4	0.0431 (11)	0.0282 (9)	0.0289 (9)	−0.0011 (8)	0.0058 (8)	0.0009 (7)

### Geometric parameters (Å, °)

**Table:**

O2—C8	1.210 (3)	C7—H7A	0.9900
O1—C5	1.245 (3)	C7—H7B	0.9900
N1—C1	1.359 (3)	C3—C2	1.395 (4)
N1—C4	1.384 (3)	C3—H3A	0.9500
N1—C9	1.466 (3)	C9—C10	1.516 (4)
O3—C8	1.323 (3)	C9—H9A	0.9900
O3—H3	0.8400	C9—H9B	0.9900
N2—C5	1.335 (3)	C1—C2	1.370 (4)
N2—C6	1.452 (3)	C1—H1	0.9500
N2—H2	0.8800	C10—H10A	0.9800
C4—C3	1.388 (3)	C10—H10B	0.9800
C4—C5	1.473 (3)	C10—H10C	0.9800
C6—C7	1.515 (3)	C2—H2A	0.9500
C6—H6A	0.9900	O4—H4A	0.96 (3)
C6—H6B	0.9900	O4—H4B	0.81 (3)
C7—C8	1.504 (3)		
			
C1—N1—C4	108.54 (19)	O2—C8—O3	123.0 (2)
C1—N1—C9	123.4 (2)	O2—C8—C7	124.0 (2)
C4—N1—C9	128.08 (19)	O3—C8—C7	113.00 (19)
C8—O3—H3	109.5	C4—C3—C2	107.7 (2)
C5—N2—C6	123.23 (18)	C4—C3—H3A	126.1
C5—N2—H2	118.4	C2—C3—H3A	126.1
C6—N2—H2	118.4	N1—C9—C10	113.3 (2)
N1—C4—C3	107.2 (2)	N1—C9—H9A	108.9
N1—C4—C5	123.25 (19)	C10—C9—H9A	108.9
C3—C4—C5	129.3 (2)	N1—C9—H9B	108.9
N2—C6—C7	111.60 (19)	C10—C9—H9B	108.9
N2—C6—H6A	109.3	H9A—C9—H9B	107.7
C7—C6—H6A	109.3	N1—C1—C2	109.2 (2)
N2—C6—H6B	109.3	N1—C1—H1	125.4
C7—C6—H6B	109.3	C2—C1—H1	125.4
H6A—C6—H6B	108.0	C9—C10—H10A	109.5
O1—C5—N2	122.0 (2)	C9—C10—H10B	109.5
O1—C5—C4	121.9 (2)	H10A—C10—H10B	109.5
N2—C5—C4	116.13 (18)	C9—C10—H10C	109.5
C8—C7—C6	112.51 (19)	H10A—C10—H10C	109.5
C8—C7—H7A	109.1	H10B—C10—H10C	109.5
C6—C7—H7A	109.1	C1—C2—C3	107.3 (2)
C8—C7—H7B	109.1	C1—C2—H2A	126.3
C6—C7—H7B	109.1	C3—C2—H2A	126.3
H7A—C7—H7B	107.8	H4A—O4—H4B	108 (3)
			
C1—N1—C4—C3	0.7 (2)	N2—C6—C7—C8	−174.39 (18)
C9—N1—C4—C3	179.4 (2)	C6—C7—C8—O2	−19.0 (3)
C1—N1—C4—C5	175.90 (19)	C6—C7—C8—O3	161.00 (19)
C9—N1—C4—C5	−5.3 (3)	N1—C4—C3—C2	−0.3 (3)
C5—N2—C6—C7	−106.2 (2)	C5—C4—C3—C2	−175.1 (2)
C6—N2—C5—O1	0.6 (3)	C1—N1—C9—C10	101.2 (3)
C6—N2—C5—C4	−179.44 (19)	C4—N1—C9—C10	−77.4 (3)
N1—C4—C5—O1	3.2 (3)	C4—N1—C1—C2	−0.8 (3)
C3—C4—C5—O1	177.3 (2)	C9—N1—C1—C2	−179.6 (2)
N1—C4—C5—N2	−176.8 (2)	N1—C1—C2—C3	0.6 (3)
C3—C4—C5—N2	−2.6 (3)	C4—C3—C2—C1	−0.2 (3)

### Hydrogen-bond geometry (Å, °)

**Table:**

*D*—H···*A*	*D*—H	H···*A*	*D*···*A*	*D*—H···*A*
O3—H3···O4^i^	0.84	1.83	2.669 (3)	173
N2—H2···O4^ii^	0.88	2.28	3.091 (3)	154
O4—H4A···O1^iii^	0.96 (3)	1.79 (3)	2.737 (2)	170 (3)
O4—H4B···O2^iv^	0.81 (3)	2.08 (4)	2.863 (3)	164 (3)

Symmetry codes: (i) *x*−1, *y*, *z*; (ii) −*x*+1, −*y*+1, −*z*+1; (iii) −*x*+1, −*y*+1, −*z*; (iv) −*x*, −*y*+1, −*z*.

## References

[bb1] Banwell, M. G., Hamel, E., Hockless, D. C. R., Verdier-Pinard, P., Willis, A. C. & Wong, D. J. (2006). *Bioorg. Med. Chem.* **14**, 4627–4638. 10.1016/j.bmc.2006.02.018 16510287

[bb2] Bruker (1999). *SMART* and *SAINT-Plus* Bruker AXS Inc., Madison, Wisconsin, USA.

[bb3] Liu, J. F., Guo, S. P. & Jiang, B. (2005). *Chin. J. Org. Chem.* **25**, 788–799.

[bb4] Liu, P.-R., Zeng, X.-C. & Xu, S.-H. (2006). *Acta Cryst.* E**62**, o1181–o1183.

[bb5] Sheldrick, G. M. (1996). *SADABS* University of Göttingen, Germany.

[bb6] Sheldrick, G. M. (2008). *Acta Cryst.* A**64**, 112–122. 10.1107/S0108767307043930 18156677

[bb7] Sosa, A. C. B., Yakushijin, K. & Horne, D. A. (2002). *J. Org. Chem.* **67**, 4498–4500. 10.1021/jo020063v 12076147

[bb8] Tang, G. H., Li, D. D., Zeng, X. C., Dong, S. S. & Wang, Y. S. (2008). *Acta Cryst.* E**64**, o1867. 10.1107/S1600536808027451 PMC2959470 21201083

[bb9] Zeng, X.-C., Xu, S.-H., Liu, P.-R. & Gu, J. (2005). *Acta Cryst.* E**61**, o1076–o1078.

